# The psychometric properties of three self-report screening instruments for identifying frail older people in the community

**DOI:** 10.1186/1471-2458-10-176

**Published:** 2010-03-31

**Authors:** Silke F Metzelthin, Ramon Daniëls, Erik van Rossum, Luc de Witte, Wim JA van den Heuvel, Gertrudis IJM Kempen

**Affiliations:** 1 Department of Health Care and Nursing Science, School for Public Health and Primary Care (CAPHRI), Faculty of Health, Medicine and Life Sciences, Maastricht University, Maastricht, The Netherlands; 2 Centre of Research on Autonomy and Participation for Chronically Ill, Zuyd University of Applied Sciences, Heerlen, The Netherlands; 3 Faculty of Health and Care, Zuyd University of Applied Sciences, Heerlen, The Netherlands; 4 Centre of Research on Technology in Care, Zuyd University of Applied Sciences, Heerlen, The Netherlands; 5 Department of Health Sciences, University Medical Centre Groningen, Groningen, The Netherlands

## Abstract

**Background:**

Frailty is highly prevalent in older people. Its serious adverse consequences, such as disability, are considered to be a public health problem. Therefore, disability prevention in community-dwelling frail older people is considered to be a priority for research and clinical practice in geriatric care. With regard to disability prevention, valid screening instruments are needed to identify frail older people in time. The aim of this study was to evaluate and compare the psychometric properties of three screening instruments: the Groningen Frailty Indicator (GFI), the Tilburg Frailty Indicator (TFI) and the Sherbrooke Postal Questionnaire (SPQ). For validation purposes the Groningen Activity Restriction Scale (GARS) was added.

**Methods:**

A questionnaire was sent to 687 community-dwelling older people (≥ 70 years). Agreement between instruments, internal consistency, and construct validity of instruments were evaluated and compared.

**Results:**

The response rate was 77%. Prevalence estimates of frailty ranged from 40% to 59%. The highest agreement was found between the GFI and the TFI (Cohen's kappa = 0.74). Cronbach's alpha for the GFI, the TFI and the SPQ was 0.73, 0.79 and 0.26, respectively. Scores on the three instruments correlated significantly with each other (GFI - TFI, r = 0.87; GFI - SPQ, r = 0.47; TFI - SPQ, r = 0.42) and with the GARS (GFI - GARS, r = 0.57; TFI - GARS, r = 0.61; SPQ - GARS, r = 0.46). The GFI and the TFI scores were, as expected, significantly related to age, sex, education and income.

**Conclusions:**

The GFI and the TFI showed high internal consistency and construct validity in contrast to the SPQ. Based on these findings it is not yet possible to conclude whether the GFI or the TFI should be preferred; data on the predictive values of both instruments are needed. The SPQ seems less appropriate for postal screening of frailty among community-dwelling older people.

## Background

Frailty is highly prevalent in older people. Up to 40% of older people can be considered as frail and an increasing trend can be expected [1]. Next to its high prevalence, frailty is characterized by its seriousness as it is related to an increased risk of adverse health outcomes such as disability [2-4]. Disability is defined as difficulty or dependency in the execution of activities of daily living and it is associated with increased health service utilization and related costs. Frailty and disability are separate but overlapping concepts. On the one hand, frailty predicts disability. On the other hand, disability may well exacerbate frailty [5]. With regard to a growing frail population and limited health care expenditures, disability in community-dwelling frail older people is suggested to be a public health problem [3]. Therefore disability prevention in community-dwelling frail older people is considered to be a priority for research and clinical practice in geriatric care [6].

Several authors emphasize a two-step approach in preventive interventions for community-dwelling frail older people, in which screening is followed by extensive assessment. With valid (screening) instruments to identify frail older people this approach may avoid costs and the unnecessary assessment of healthy people [7,8]. During the last few decades, various instruments, based on various definitions, have been developed to detect frailty. This has lead to a diversity of prevalence estimates of frailty [4]. Little is yet known about the reliability and validity of these instruments and no gold standard exists. Therefore, more insight into the psychometric properties of frailty instruments is relevant for geriatric care and research in this area [4].

Frailty instruments have been developed from the point of view of different perspectives on frailty [9]. From a physiological perspective physical frailty markers, such as unintentional weight loss or weakness (grip strength), are used to identify frail older people [5]. Next to physical factors, a multifactorial perspective on frailty also takes psychological, social and environmental factors into account [10]. An example of such an instrument is the Frailty Index [11,12], which combines, for example, physical frailty markers such as weight loss and grip strength with other factors such as cognition, mood or limitations in (instrumental) activities of daily living. Frailty may be elaborated more sharply if it is described from a physiological perspective, however, the usefulness of this perspective in daily practice is questioned, as frailty cannot be separated from other factors such as cognition, mood or social support [9].

Frailty instruments can be divided into self-report and performance-based instruments [9]. It is assumed that performance-based instruments provide more precise and valid answers [13,14]. Although they are less influenced by socio-demographic variables, personality and cognitive and affective factors, they are more sensitive to non-response, changes in time and differences in the execution of activities. Furthermore, they are less easy to conduct and time-consuming [13-15]. Self-report measures are believed to be an efficient method for reaching large groups and for providing high response rates and reliable and valid answers [15].

In this study, we present the psychometric properties of frailty instruments that define frailty from a multifactorial perspective and are applicable for postal screening of community-dwelling older people. Given this objective and the target population, the Groningen Frailty Indicator (GFI) [16], the Tilburg Frailty Indicator (TFI) [17] and the Sherbrooke Postal Questionnaire (SPQ) [8] were chosen. The GFI and the SPQ have been used in previous studies for the purpose of postal screening [18-22], however, empirical evidence about the psychometric properties of the GFI, the TFI, and the SPQ is still scarce. The purpose of the present study was to evaluate and compare their psychometric properties.

## Methods

### Study design and participants

A cross-sectional study was conducted in a sample of 687 community-dwelling older people living in the areas of Limburg and Utrecht in the Netherlands. Older people were identified between November 2008 and April 2009 from the panels of three general practitioners (GPs). All persons aged 70 years or above from each of the panels were invited by these GPs to participate in the study and to fill in a short questionnaire. The questionnaire included the three frailty instruments (GFI, TFI, SPQ) and an instrument that measures disability with respect to (instrumental) activities of daily living: the Groningen Activity Restriction Scale (GARS) [23]. After two weeks a reminder was sent to non-respondents. Participants of the study were well informed about the study in a patient information letter that accompanied the questionnaire. The information letter was formulated according to the guidelines of good clinical practice. Participants had to give written informed consent. The study did not require ethical approval. The postal procedure, including the questionnaire, has proven to be feasible for postal screening in a pilot study [24].

### Data collection

The three frailty instruments and the disability measure are briefly described below. For an overview of all items please see Additional file 1: *Frailty Instruments: Overview of all items*.

### Measures

The Groningen Frailty Indicator (GFI), developed by Steverink and colleagues [16], is a screening instrument for determining the level of frailty. It consists of fifteen items and focuses on the loss of functions and resources in four domains of functioning: physical (nine items), cognitive (one item), social (three items) and psychological (two items). Most items can be answered with 'yes'or 'no'. For the cognitive and psychosocial items the option 'sometimes' is added. Scores on the GFI range from zero to fifteen. A total score of four or higher is considered as moderately to severely frail [16,21]. A study by Steverink and colleagues [16] suggested that the GFI is an internally consistent scale with positive indications for construct and clinical validity.

The Tilburg Frailty Indicator (TFI) has recently been described by Gobbens and colleagues [17] and consists of two subscales. The first subscale (ten items) comprises determinants of frailty, for example, socio-demographic data and data about life-events and chronic diseases. Socio-demographic data (age, sex, educational level and income) were used for validation purposes. The analyses of psychometric properties focus on the second subscale, which determines the level of frailty. This subscale consists of fifteen items that are about physical (eight items), social (three items) and psychological factors (four items), including one item which is about cognition. Most items can be answered with 'yes'or 'no'. For the psychological items the option 'sometimes' is added. Scores for the TFI range from zero to fifteen. A score of five or higher is considered to be associated with frailty [17].

The Sherbrooke Postal Questionnaire (SPQ) was developed by Hébert and colleagues [8] and consists of six items aiming to identify frail older people in the community. The items focus on the physical (four items), social (one item) and cognitive (one item) domains of functioning. Items can be answered with 'yes'or 'no'. Scores range from zero to six. Those older persons scoring two or higher, or who do not respond to the questionnaire, are considered to have an increased risk for functional decline and therefore are assumed to be frail. It should be noted that in the present study non-respondents were excluded from the analyses. In a Canadian sample of community-dwelling older people, predictive validity with regard to functional decline has been found [8]. There are also indications for its predictive validity with regard to requirements for further assessment [20], use of emergency services [22] and mortality [19].

The Groningen Activity and Restriction Scale (GARS) [23] is a valid and reliable instrument and consists of two subscales. The first subscale is about activities of daily living (ADL) (eleven items). The second subscale relates to instrumental activities of daily living (IADL) (seven items). Items can be answered on a four point scale ranging from 'Yes, I can do it fully independently without any difficulty'to 'No, I cannot do it fully independently; I can only do it with someone's help'. Scores range from 18 to 72 (total scale), from 11 to 44 (ADL subscale) and from 7 to 28 (IADL subscale). Higher scores indicate greater disability in activities of daily living.

### Statistical analysis

First, to provide an overview of respondents' background characteristics, descriptive statistics were used.

Secondly, the reliability was determined from agreement between instruments (Kappa statistic based on proposed cut-off points by original authors) and internal consistency. Cronbach's alpha coefficient was calculated to evaluate internal consistency of items. Cronbach's alpha produces the same result as the Kuder-Richardson Formula 20 (K-R-20), which can be used to assess the internal consistency for dichotomous items [25]. Furthermore, corrected total-item-correlations were calculated.

Thirdly, to assess the validity, non-parametric tests were used as our data were not normally distributed. If less than 25% (GFI, TFI, SPQ) or 50% (GARS) [23] of the items were missing, these were imputed by means of case mean substitution [26]. If more items were missing, persons were excluded from the analysis for the particular scale. The construct validity was assessed using Spearman's rank correlation between the three frailty instruments, as the instruments were assumed to measure the same concept of frailty. Frailty and disability are strongly related concepts [5], as frail older people have an increased risk of disability and disability exacerbates frailty [2,3,5]. Substantial associations between frailty and disability were expected. Therefore, construct validity was also assessed by examining associations between frailty and disability, measured by means of the GARS (Spearman's rank correlation). However, correlations should not be too high, otherwise frailty instruments and the GARS would measure the same concept. Furthermore, frail older people were more likely to be older, female, less educated, and had lower incomes compared to their non-frail counterparts [27]. Since the distribution of frailty scores was non-normally distributed, Mann-Whitney U and Kruskal-Wallis tests were performed to evaluate differences in the distribution of frailty scores among groups with different background characteristics [28]. For the dichotomous variable gender (female versus male) the Mann-Whitney U test was applied. For categorical variables with more than two groups (age, education and income), Kruskal-Wallis tests were used to compare the distribution of frailty scores among groups.

All statistical analyses were performed using SPSS for Windows, version 16.0. The level of statistical significance was set at p = 0.05 (two-tailed). For post-hoc pairwise comparisons a Bonferroni correction was applied, so all effects are reported at a p = 0.02 level of significance (two-tailed) in the case of three groups (age, education, income).

## Results

### Participants

Of the 687 community-dwelling older people (≥70 years), 532 (77.4%) returned the questionnaire. The sample consisted of 311 women (58.5%) and 221 men (41.5%). In total, 64% of respondents lived in an urban area (Roermond, Amersfoort), while 36% lived in a rural area (Roggel). When using the proposed cut-off points, the GFI detected 245 frail cases (46.3%). The TFI and the SPQ identified 211 (40.2%) and 305 (59.1%) frail older people, respectively. The mean age of respondents was 77.2 years with a range of 70-97 years (SD = 5.5). Nearly half of the sample (48.6%) had a secondary educational level. The largest proportion of people (42.4%) had a net income of more than €1500 (per month/per household). An overview of background characteristics is presented in Table 1. The sample is representative for the Dutch population of older people. According to a report of the Netherlands Institute for Social Research [29] slightly more people aged 75 years and older are female. Older people, especially women, are often less educated and have an average income of about €1500 (per month/per household).

**Table 1:** Characteristics of the participants (n = 532).

		Men (n = 221)	Women (n = 311)	Total (n = 532)
Frail^1^	n (%)			
GFI		86 (39.3)	159 (51.3)	245 (46.3)
TFI		66 (30.1)	145 (47.4)	211 (40.2)
SPQ		148 (67.9)	157 (52.7)	305 (59.1)
Age^2^	n (%)			
70-74 yrs		91 (41.2)	102 (32.8)	193 (36.3)
75-79 yrs		78 (35.3)	115 (37.0)	193 (36.3)
≥ 80 yrs		52 (23.5)	94 (30.2)	146 (27.4)
Education	n (%)			
No Education/Primary Education		62 (28.6)	124 (40.8)	186 (35.7)
Secondary Education		102 (47.0)	151 (49.7)	253 (48.6)
Higher Education		53 (24.4)	29 (9.5)	82 (15.7)
Income	n (%)			
≤ €900		34 (16.1)	59 (20.6)	93 (18.7)
€901 to €1500		63 (29.9)	131 (45.6)	194 (39.0)
≥ €1501		114 (54.0)	97 (33.8)	211 (42.4)

^1 ^Based on proposed cut-off points by original authors: GFI ≥ 4, TFI ≥ 5, SPQ ≥ 2.

^2 ^Mean age men 76.6 years (SD 5.4), mean age women 77.6 years (SD 5.5).

The frailty instrument with the greatest number of excluded respondents due to missing values (> 25% missing values) was the SPQ (n = 8). For the GFI and the TFI, one and two persons, respectively, were excluded due to missing values. On an item-level the number of missing values ranged from zero to eight (GFI), from zero to twelve (TFI) and from one to ten (SPQ). The average number of missing values per item was 2.4, 5.1 and 5.3 for the GFI, the TFI and the SPQ, respectively.

### Reliability

Cohen's Kappa coefficients between instruments were 0.74 (GFI - TFI), 0.28 (SPQ - GFI) and 0.25 (SPQ - TFI). According to Landis & Koch [30] the kappa values indicated good agreement between GFI and TFI and fair agreement between the between GFI and SPQ and TFI and SPQ (< 0.20 = poor, 0.21 - 0.40 = fair, 0.41 - 0.60 = moderate, 0.61 - 0.80 = good, 0.81 - 1.00 = very good agreement). Cronbach's alpha coefficients for the GFI, the TFI and the SPQ were α = 0.73, α = 0.79 and α = 0.26, respectively. The higher Cronbach's alpha, the more reliable the test is. Alpha values above 0.70 indicated a satisfactory internal consistency for a scale [31]. Corrected item-total correlations ranged from 0.14 to 0.55 with a mean of 0.30 (GFI), from 0.18 to 0.58 with a mean of 0.39 (TFI) and from 0.13 to 0.25 with a mean of 0.18 (SPQ).

### Validity

Frailty instruments correlated significantly (p < 0.05) with each other and with disability measured by means of the GARS (convergent validity). The association between the GFI and the TFI scores was r = 0.87. Correlations with the SPQ scores were r = 0.47 for the GFI and r = 0.42 for the TFI. The correlation coefficients between frailty instruments and disability (GARS) were r = 0.57 (GFI - GARS), r = 0.61 (TFI - GARS) and r = 0.46 (SPQ - GARS). An overview of all correlation coefficients is presented in Table 2.

**Table 2:** Spearman correlation coefficients (99%-confidence interval) among frailty instruments and GARS.

	GFI	TFI	SPQ	GARS Total scale	GARS ADL scale
TFI	0.87 (0.84-0.89)				
SPQ	0.47 (0.1-0.55)	0.42 (0.32-0.51)			
GARS	0.57	0.61	0.46		
Total scale	(0.49-0.64)	(0.53-0.68)	(0.37-0.54)		
GARS	0.54	0.58	0.41	0.94	
ADL scale	(0.46-0.61)	(0.5-0.65)	(0.32-0.5)	(0.93-0.95)	
GARS	0.55	0.57	0.46	0.96	0.79
HDL scale	(0.47-0.62)	(0.49-0.64)	(0.37-0.54)	(0.95-0.97)	(0.74-0.83)

Table 3 shows the mean total scores and standard deviations of the GFI, the TFI and the SPQ related to (a) age, (b) sex, (c) education and (d) income. Scores on the GFI and the TFI were significantly higher for females, for persons with a higher age and for persons with lower education and lower incomes as compared to males, persons with a lower age, and persons with higher education and higher incomes. In contrast, on the SPQ we found higher scores among males as compared to females. Scores on the SPQ increased with higher age, lower education and lower incomes, however, the differences with respect to education and income were not significant (p = 0.29 and p = 0.08 respectively).

**Table 3:** Mean scores on frailty instruments ^1 ^according to sex, age, educational level and income.

		GFI	TFI	SPQ
Total sample	Mean (sd)	3.6 (2.8)	4.2 (3.2)	1.9 (1.2)

Sex	Mean (sd)			
Male		3.2 (2.7)	3.4 (3.1)	2.1 (1.2)
Female		3.9 (2.8)	4.7 (3.2)	1.7 (1.2)
	Z statistic^2^ (P-value)	-3.31 (0.001)	-4.95 (0.000)	-3.28 (0.001)
Age	Mean (sd)			
≤ 74 yrs		3.0 (2.7)	3.3 (3.1)	1.7 (1.2)
75-79 yrs		3.6 (2.8)	4.2 (3.3)	1.9 (1.2)
≥ 80 yrs		4.4 (2.7)	5.3 (3.1)	2.2 (1.2)
	Chi-square^3 ^(P-value)	27.58 (0.000)	37.2 (0.000)	15.84 (0.000)
Education	Mean (sd)			
No Education/Primary Education		4.1 (3.0)	4.7 (3.5)	2.0 (1.3)
Secondary Education		3.6 (2.7)	4.1 (3.1)	1.8 (1.2)
Higher Education		2.7 (2.2)	3.1 (2.8)	1.7 (1.0)
	Chi-square^3 ^(P-value)	12.13 (0.002)	13.47 (0.001)	2.47 (0.291)
Income	Mean (sd)			
≤ €900		4.6 (3.0)	5.3 (3.6)	2.2 (1.4)
€901 to €1500		4.0 (2.9)	4.8 (3.4)	1.8 (1.3)
≥ € 1501		2.8 (2.3)	3.1 (2.6)	1.8 (1.0)
	Chi-square^3^(P-value)	29.42 (0.000)	37.16 (0.000)	5.05 (0.080)

^1 ^Higher scores indicate poorer functioning.

^2^ Mann-Whitney U test

^3 ^Kruskal Wallis test

## Discussion

The purpose of the present study was to evaluate and compare the psychometric properties of three screening instruments that define frailty from a multifactorial perspective and which are applicable for postal screening in community-dwelling frail older people. The chosen instruments were the Groningen Frailty Indicator (GFI), the Tilburg Frailty Indicator (TFI) and the Sherbrooke Postal Questionnaire (SPQ).

From the present study we may conclude that: (1) prevalence estimates of frailty ranged between 40.2% (TFI), 46.3% (GFI) and 59.1% (SPQ); (2) the agreement in identifying frailty between the GFI and the TFI was satisfactory (kappa = 0.74) and the agreements between the SPQ and the GFI and the TFI, respectively, were much lower; (3) both the GFI and the TFI had high internal consistency in contrast to the SPQ; (4) the GFI and the TFI had better construct validity in comparison with the SPQ.

Prevalence estimates of 40% to 60% found in the present study can be considered as high. It is important to bear in mind that prevalence estimates strongly depend on the interpretation of the concept of frailty and the approach that is chosen to measure it [32]. In a recent study by Santos-Eggimann and colleagues [33], a distinction was made between frail and pre-frail older people based on the frailty phenotype of Fried and colleagues [5,34]. In a Dutch sample of community-dwelling older people, Santos-Eggimann and colleagues [33] found a frailty prevalence of 11.3%, while 38.5% were identified as pre-frail. These results indicate that the instruments in our study, based on the proposed cut-off points, may identify pre-frail instead of frail older people. Further research is needed to provide a better view on relevant cut-off points for frailty instruments. Longitudinal studies are needed to investigate the predictive power of instruments to identify older people who are at risk for adverse health outcomes in the near future.

Steverink and colleagues [16] suggested that the GFI is an internally consistent scale with positive indications for construct and clinical validity. The present study supports these findings. Similar results for the TFI may be explained by seven out of fifteen items of the TFI being identical with the GFI. These items are about hearing and vision capacity, unintentional weight loss and psychosocial and cognitive functioning. Please see Additional file 1: *Frailty Instruments: Overview of all items *for more information about the instruments. Scores on the Sherbrooke Postal Questionnaire were higher for males compared with females. This finding is inconsistent with the literature [27]. However, other findings on the Sherbrooke Questionnaire (higher score with higher age, lower educational level and lower incomes) are well in line with the literature [27]. Previous studies about the SPQ have reported positive results regarding the predictive validity of the SPQ [8,19,20,22], however, in the present study the SPQ showed less reliability and construct validity. Conclusions about predictive validity can not be drawn for any of the three instruments.

The findings of the present study should be interpreted in the context of potential limitations. First, little is known about the test-retest reliability of the instruments. Second, there is no gold standard available as an external criterion of frailty. Future studies could analyse the predictive validity of the frailty instruments with respect to disability, health service utilization and mortality. Last, the SPQ was not fully used according to the protocol, as non-respondents were excluded from analyses. According to the protocol of the SPQ [8], non-respondents should also be considered at risk (which would have resulted in a prevalence estimate of 67.0% instead of 59.1%). The strengths of the present study are the comparisons of the psychometric properties of the frailty instruments, the proven feasibility of the postal procedure [24] and the response rate of 77.4%, which is as good as, or even better than, previous studies in which postal screening procedures were applied [24,35,36].

Although most older people may visit their GP regularly, primary care often fails in the identification of the health care needs of older people [37]. Screening has the potential to identify older people at risk, followed by comprehensive assessment when needed [7,8]. Frailty instruments have to provide reliable and valid answers and have to be feasible [15]. The psychometric properties of the TFI were slightly better than those of the GFI. However, the number of missing values was lower for GFI items than for TFI items, indicating a higher feasibility of the GFI. Based on these findings it is not yet possible to conclude whether the GFI or the TFI should be preferred for postal screening. The SPQ is less appropriate with regard to its psychometric quality and missing values.

The frailty index [8,11] is a simple measure that is based on self-reports. However, less is known about its feasibility for postal screening. Investigating the feasibility and validity of the frailty index as a postal screening instrument may be a point of interest for future research.

Future (longitudinal) research into the psychometric properties of the GFI and the TFI is urgently needed with regard to predictive validity and test-retest reliability of the GFI and the TFI. In addition, comparing the GFI and the TFI with other frailty-related constructs would lead to more insight into their construct validity.

## Conclusion

Valid screening instruments for identifying community-dwelling frail older people are needed for disability prevention. The GFI and the TFI have shown high internal consistency and construct validity, in contrast to the SPQ. Prevalence estimates of frailty ranged from 40% to 59%. Most agreement was found between the GFI and the TFI. Based on these findings, it is not possible to conclude whether the GFI or the TFI should be preferred for screening. The SPQ seems less appropriate. Further research is needed.

## Abbreviations

GFI: Groningen Frailty Indicator; TFI: Tilburg Frailty Indicator; SPQ: Sherbrooke Postal Questionnaire; GARS: Groningen Activity Restriction Scale; GP: General practitioner; ADL: activities of daily living; IADL: instrumental activities of daily living.

## Competing interests

The authors declare that they have no competing interests.

## Authors' contributions

SFM and RD designed the study and collected the data. SFM analysed the data and drafted the manuscript with contributions of RD, EvR, LdW, WJAvdH and GIJMK. All authors read and approved the final manuscript.

## Pre-publication history

The pre-publication history for this paper can be accessed here:

http://www.biomedcentral.com/1471-2458/10/176/prepub

## Supplementary Material

Additional file 1 **Frailty Instruments: Overview of all items**. The file contains an overview of all items of the Groningen Frailty Indicator, the Tilburg Frailty Indicator (2^nd ^subscale) and the Sherbrooke Postal Questionnaire. Click here for file
