# Supplementary material for: The psychometric properties of three self-report screening instruments for identifying frail older people in the community
Source: BMC Public Health. 2010 Mar 31;10:176. doi: 10.1186/1471-2458-10-176 (PMC2868816; doi:10.1186/1471-2458-10-176)
Supplement: Additional file 1 — Frailty Instruments: Overview of all items. The file contains an overview of all items of the Groningen Frailty Indicator, the Tilburg Frailty Indicator (2nd subscale) and the Sherbrooke Postal Questionnaire. [file 1471-2458-10-176-S1.PDF]

Additional files

Additional file 1 – Frailty Instruments: Overview of all items

The Groningen Frailty Indicator (GFI)

	0	1
**Physical components**		
Are you able to carry out these tasks single-handedly and without any help? (The use of help resources such as a walking stick, walking frame or wheelchair is considered to be independent.)		
1. Shopping		
2. Walking around outside (around the house or to the neighbours)		
3. Dressing and undressing		
4. Going to the toilet		
5. What mark do you give yourself for physical fitness? (scale 0 to 10)		
6. Do you experience problems in daily life due to poor vision?		
7. Do you experience problems in daily life due to being hard of hearing?		
8. During the last 6 months have you lost a lot of weight unwillingly? (3 kg in 1 month or 6 kg in 2 months)		
9. Do you take 4 or more different types of medicine?		
**Cognitive component**		
10. Do you have any complaints about your memory?		
**Social component**		
11. Do you sometimes experience emptiness around yourself?		
12. Do you sometimes miss people around yourself?		
13. Do you sometimes feel abandoned?		
**Psychological component**		
14. Have you recently felt downhearted or sad?		
15. Have you recently felt nervous or anxious?		
**Scoring:**		
Questions 1-4: Yes = 0; No = 1		
Question 5: 0-6 = 1; 7-10 = 0		
Questions 6-9: No = 0; Yes = 1		
Question 10: No = 0; Sometimes = 0; Yes = 1		
Questions 11-15: No = 0; Sometimes = 1; Yes = 1		

The Tilburg Frailty Indicator (TFI) (2nd subscale)

	0	1

Physical component

1. Do you feel healthy?
2. Have you lost a lot of weight recently without wishing to do so? (‘A lot’ is 6 kg or more during the last six months or 3 kg or more during the last month.)

Do you experience problems in your daily life due to:

3. Difficulty in walking?
4. Difficulty with maintaining your balance?
5. Poor hearing?
6. Poor vision?
7. Lack of strength in your hands?
8. Physical tiredness?

Psychological component

9. Do you have problems with your memory?
10. Have you felt down during the last month?
11. Have you felt nervous or anxious during the last month?
12. Are you able to cope with problems well?

Social component

13. Do you live alone?
14. Do you sometimes wish you had more people around you?
15. Do you receive enough support from other people?

Scoring:

Question 1: Yes = 0; No = 1
Questions 2-8: No = 0; Yes = 1
Question 9: No = 0; Sometimes = 0; Yes = 1
Questions 10-11: No = 0; Sometimes = 1; Yes = 1
Question 12: No = 1; Yes = 0
Question 13: No = 0; Yes = 1
Question 14: No = 0; Sometimes = 1; Yes = 1
Question 15: Yes = 0; No = 1

The Sherbrooke Postal Questionnaire (SPQ)

		0	1
1.	Do you live alone?		
2.	Do you take more than three different medications every day?		
3.	Do you regularly use a cane, a walker or a wheelchair to move about?		
4.	Do you see well?		
5.	Do you hear well?		
6.	Do you have problems with your memory?		

Scoring:

Question 1: Yes = 0; No = 1
Questions 2-3: No = 0; Yes = 1
Questions 4-5: Yes = 0; No = 1
Question 6: No = 0; Yes = 1
